# Can cognition help predict suicide risk in patients with major depressive disorder? A machine learning study

**DOI:** 10.1186/s12888-022-04223-4

**Published:** 2022-09-01

**Authors:** Shuqiong Zheng, Weixiong Zeng, Qianqian Xin, Youran Ye, Xiang Xue, Enze Li, Ting Liu, Na Yan, Weiguo Chen, Honglei Yin

**Affiliations:** 1 Department of Psychiatry, Nanfang Hospital, Southern Medical University, Guangzhou, China; 2 Guangdong-Hong Kong-Macao Greater Bay Area Center for Brain Science and Brain-Inspired Intelligence, Guangzhou, China; 3 Department of Radiology, Nanfang Hospital, Southern Medical University, Guangzhou, China

**Keywords:** Cognition, Machine learning, Suicide attempt, Depression

## Abstract

**Background:**

Previous studies suggest that deficits in cognition may increase the risk of suicide. Our study aims to develop a machine learning (ML) algorithm-based suicide risk prediction model using cognition in patients with major depressive disorder (MDD).

**Methods:**

Participants comprised 52 depressed suicide attempters (DSA) and 61 depressed non-suicide attempters (DNS), and 98 healthy controls (HC). All participants were required to complete a series of questionnaires, the Suicide Stroop Task (SST) and the Iowa Gambling Task (IGT). The performance in IGT was analyzed using repeated measures ANOVA. ML with extreme gradient boosting (XGBoost) classification algorithm and locally explanatory techniques assessed performance and relative importance of characteristics for predicting suicide attempts. Prediction performances were compared with the area under the curve (AUC), decision curve analysis (DCA), and net reclassification improvement (NRI).

**Results:**

DSA and DNS preferred to select the card from disadvantageous decks (decks "A" + "B") under risky situation (*p* = 0.023) and showed a significantly poorer learning effect during the IGT (F = 2.331, *p* = 0.019) compared with HC. Performance of XGBoost model based on demographic and clinical characteristics was compared with that of the model created after adding cognition data (AUC, 0.779 vs. 0.819, *p* > 0.05). The net benefit of model was improved and cognition resulted in continuous reclassification improvement with NRI of 5.3%. Several clinical dimensions were significant predictors in the XGBoost classification algorithm.

**Limitations:**

A limited sample size and failure to include sufficient suicide risk factors in the predictive model.

**Conclusion:**

This study demonstrate that cognitive deficits may serve as an important risk factor to predict suicide attempts in patients with MDD. Combined with other demographic characteristics and attributes drawn from clinical questionnaires, cognitive function can improve the predictive effectiveness of the ML model. Additionally, explanatory ML models can help clinicians detect specific risk factors for each suicide attempter within MDD patients. These findings may be helpful for clinicians to detect those at high risk of suicide attempts quickly and accurately, and help them make proactive treatment decisions.

**Supplementary Information:**

The online version contains supplementary material available at 10.1186/s12888-022-04223-4.

## Background

Despite major advances in the treatment of mental health problems over recent decades, suicide rates remain high [1]. The World Health Organization estimated that there are 800,000 suicides per year worldwide, and 20–40 times more non-fatal suicide attempts [2]. This discrepancy may highlight the importance of better understanding the risk factors for suicide [3]. The prediction of suicide attempts is a complex classification problem requiring the simultaneous consideration of tens, or even hundreds of risk factors. Any risk factor considered in isolation will be an inaccurate predictor [2]. Thus, more comprehensive studies are needed on the mechanisms of suicides.

Psychiatric disorders, especially major depressive disorder (MDD), have the strongest effect on suicide rates [2]. Cognitive deficits represent a core feature of patients with MDD, and may be a predisposing factor for suicide attempts [4]. Cognitive deficits have been proposed as candidate "endophenotypes" for research on the genetics of suicide [5]. The cognitive deficits of suicidal individuals are characterized by "cognitive rigidity" [6]. In MDD with and without suicidal behavior, deficits in decision-making and processing speed have been observed in several studies [7, 8]. Furthermore, deficits of decision-making may increase the risk of suicide above and beyond the influence of depression [9].

Compared to healthy controls, decision-making tasks are poorly performed by patients with a history of suicide attempts, indicating that impaired decision-making may be a neuropsychological risk factor for suicidal behavior [10, 11]. The Iowa Gambling Task (IGT) has been used to study decision-making in various clinical populations and appears well-suited to characterize cognitive deficits in suicidal individuals [12]. Studies found that suicidal individuals perform worse than healthy controls [10]. On this basis, we might suggest that serious cognitive deficits, such as poor decision-making abilities, potentially increase the risk of suicide.

As risk factors could be combined in a complex but replicable manner, we recommend that studies focused on prediction of suicide attempts prioritize the development of risk algorithms, using tools such as machine learning (ML) approaches [13].

As a subfield of artificial intelligence, ML is an approach to build optimal predictive models by processing complex relationships in data. It has been applied to the field of suicide science [14]. Some ML algorithms have been developed to build suicide predictive models, using methods such as Decision Tree, Support Vector Machine, Random Forest, Naïve Bayes, and Artificial Neural Network [15, 16]. The current suicide attempt predictive models were developed mostly based on demographics, clinical information, and biological variables [17]. Most models recognized that childhood trauma [18, 19], impulsivity, and aggression [2, 20] were risk factors for suicide attempts [21].

Although the predictive models from previous studies have achieved high accuracy in classifying suicide attempts and non-attempts, few studies focus on the association between cognitive function and suicide attempts. In this study, therefore, we hypothesize that the decision-making function of depressed suicide attempters (DSA) may be different from depressed non-suicide attempters (DNS) and construct a predictive model for suicide attempts among patients with MDD. In this predictive model that can effectively identify DSA, in addition to multiple questionnaire assessments, we emphasize cognitive function as critical predictors.

## Methods

### Participants

Patients (*N* = 113, aged 18–65 years old) with MDD were recruited from the population of psychiatric inpatients and outpatients at Nanfang Hospital (Guangzhou, China). Diagnosis was established using the Chinese-Bilingual Structured Clinical Interview for the Diagnostic and Statistical Manual of Mental Disorders, 4th Edition (Axis I, Patient Version) (CB-SCID-I/P). Patients with a Hamilton Depression Rating Scale (HAMD-24) score > 20 were recruited. The depressive symptoms of patients persist for at least 2 weeks. The exclusion criteria for patients were (a) a history of neurological disease and the presence of psychiatric disorders on either Axis I (e.g. schizophrenia spectrum) or Axis II (personality disorders, mental retardation, etc.), (b) co-morbid substance use disorders, (c) using drugs including mood stabilizers, antidepressants, anxiolytics, antipsychotic and benzodiazepines within the previous two weeks, (d) severe somatic illness, (e) a history of manic episodes, and (f) inability to provide informed consent. A healthy volunteer group (*N* = 98) was recruited through advertisements. These controls were evaluated by psychiatrists for the presence of possible psychiatric diagnoses, personality disorders, and suicidal behaviors, using the Non-patient Edition of CB-SCID-I (CB-SCIDI/NP). The controls were excluded if they had (a) a diagnosis of any psychiatric disorder, (b) personal history of suicidal thoughts or attempted suicide, and (c) severe neurological or somatic disease.

All participants were interviewed by experienced clinical psychiatrists. Demographic data, including gender, age, educational level, and clinical information, including the history of suicidality, the severity of depression, and cognitive function (decision-making and attentional bias), were recorded using a series of scales and neuropsychological tasks.

A total of 52 suicide attempters with MDD (DSA, 12 men, 23.1% and 40 women, 76.9%) and 61 non-attempterswith MDD (DNS, 25 men, 41.0% and 36 women, 59.0%), and 98 health controls (HC, 49 men, 50% and 49 women, 50%) were enrolled in the study. Younger subjects were found in the DSA group, compared to the HC and DNS (Table 1).

**Table 1:** Demographic and clinical features, as well as comparison results without adjusting covariates

Items		DSA* (*N* = 52)	DNS* (*N* = 61)	HC* (*N* = 98)	F/*x*^2^	Significant direction
		**Mean (sd) / N (%)**	**Mean (sd) / N (%)**	**Mean (sd) / N (%)**	**(*p*-value)**	
Age		25.54 (8.55)	29.87 (11.50)	29.56 (7.11)	4.160	DSA < DNS、 HC
					(0.017)	
Gender	Male	12 (23.1%)	25 (41.0%)	49 (50.0%)	10.152	DSA: male < female
	Female	40 (76.9%)	36 (59.0%)	49 (50.0%)	(0.006)	
Education level	≥ 12 years	22 (42.3%)	31 (50.1%)	47 (48.0%)	0.839 (0.657)	
	< 12 years	30 (57.7%)	30 (49.9%)	51 (52.0%)		
suicide attempt lethality	High-lethality	9(17.3%)	\	\	\	\
	low-lethality	43(82.7%)				
Suicide attempt recency	Within one week	9 (17.3%)	\	\	\	\
	Not within one week	43 (82.7%)				
IGT* performance						
Net total score		-13.73 (28.883)	-16.53 (33.422)	-10.06 (34.148)	0.755(0.471)	
PartA(Trails 1–40)		-4.54 (12.221)	-6.27 (13.956)	-9.35 (12.315)	2.666(0.072)	
PartB(Trails 41–100)		-9.17 (19.571)	-10.03 (20.881)	-0.735 (25.015)	3.838(0.023)	HC > DSAˎDNS
Block1(Trials 1–20)		-3 (6.669)	-3.8 (7.269)	-5.27 (6.725)	2.056(0.131)	
Block2(Trials 21–40)		-1.54 (7.868)	-2.47 (8.848)	-4.08 (7.896)	1.818(0.165)	
Block3(Trials 41–60)		-2.38 (7.118)	-4.07 (9.072)	-1.55 (9.079)	1.583(0.208)	
Block4(Trials 61–80)		-3.94 (7.092)	-3.67 (8.471)	-0.51 (9.465)	3.782(0.024)	HC > DSAˎDNS
Block5(Trials 81–100)		-2.85 (9.712)	-2.34 (9.345)	1.33 (9.877)	4.261(0.015)	HC > DSAˎDNS
SST* performance						
positiveRT		726.51(261.209)	690.991(240.908)	\	-0.751(0.454)	
negativeRT		741.257(256.722)	686.08(246.231)	\	-1.164(0.247)	
neutralRT		743.001(243.265)	670.407(235.604)	\	-1.608(0.111)	
suicideRT		756.389(281.243)	698.087(253.441)	\	-1.159(0.249)	
"suicide"word RT		756.81(361.531)	684.317(297.816)	\	-1.153(0.251)	
HAMD-24*		36.212(6.241)	29.016(6.828)	\	-5.807(0)	DSA > DNA
CTQ*score		53.94(12.299)	47.33(14.048)	\	-2.640(0.009)	DSA > DNA

^*^DSA Depressed suicide attempter, *DNS* Depressed non-suicide attempter, *HC* Healthy control

*IGT* IOWA gambling task, *SST* Suicide stroop task, *HAMD-24* Hamilton Depression Scale-24 items, *CTQ* Childhood Trauma Questionnaire

Each participant signed an informed consent form, approved by the Southern Medical University Clinical Research Ethics Committee (Reference Number: NFEC-2018–041). The entire study process followed the guidelines and regulations of the Committee.

### Instruments

#### Decision-making

Decision-making was tested using the Iowa Gambling Task (IGT) [22]. All participants received standard instructions. They were told that the goal of the game was to win as much money as possible. Four decks of cards were presented on the screen, labeled as "A," "B," "C," and "D." Each deck contained 40 cards. Participants chose cards multiple times from these decks by clicking on the top card. They were informed that the decks were different from each other, and the game was fair and did not work randomly; therefore, there were advantageous and disadvantageous decks. The "A" and "B" decks were disadvantageous, associated with immediate higher rewards, but even more severe future punishments. The "C" and "D" decks were advantageous in the long term, providing moderate immediate rewards, but also moderate losses.

The tasks consist of 100 trials. For each trial, the given choices were categorized as advantageous or disadvantageous. The total net score on the IGT was calculated as the difference between the total number of card selections from advantageous decks (decks "C" + "D") and from disadvantageous decks (decks "A" + "B"). Net scores were calculated for each of 5 blocks consisting of 20 consecutive trials [11]. The net score for the first 40 and last 60 trials were also calculated to represent performance in the decision under ambiguity and decision under risk, respectively [23].

The total net score can range from -100 to 100. The range for each block score is -20 to 20. Positive total net scores and block scores indicate that decision-making performance was advantageous [24].

#### Suicide attempt

A semi-structured clinical interview about the intent and medical severity of suicidal behaviors was used to ascertain lifetime history of suicide attempts. A suicide attempt was defined as engaging in potentially self-damaging behavior with an intent to die. The behavior may result in injury or at least involve the potential for injury. Other non-suicidal self-injury (e.g., self-mutilation) or suicidal ideation without any attempt were excluded [2]. Suicidal intent can be determined by inquiring about the individual's intent for the behavior or can be inferred based on the individual’s perception of the lethality of the behavior when failed to be determined according to the individual’s answers (e.g., the individual refuses to provide relevant information) [25].

The suicide attempters subsequently were divided into two groups: High-lethality suicide attempts (HLSA, *N* = 9) and low-lethality suicide attempts (LLSA, *N* = 43), according to the following definitions of lethality [26]. A classification of HLSA was given for suicide attempts that require at least 24 h of hospitalization, or treatment in special units (including intensive care, hypertension, or burn units), general anesthesia, extensive medical treatment (except gastric lavage, activated carbon, or routine nerve observation); LLSA for suicide attempts that do not meet the above criteria.

#### Attentional bias

Attentional bias and processing speed were tested using Suicide Stroop Task (SST) [22], which records participants' response times (RT; latencies) when identifying the color of different words presented on the computer screen. Longer response times indicate greater attentional bias due to content of the presented words and lower processing speed.

Twelve positive, twelve negative, twelve neutral, and twelve suicide-related words were presented throughout the task. Each category was presented 24 times. Our previous study found slower processing speed in depressed patients, regardless of suicide attempt history. Furthermore, depressed attempters showed enhanced positive-word response time compared to depressed non-attempters [27]. Therefore, based on these previous findings, the performance of SST was no longer used for statistical analysis, but for ML in this study.

#### Clinical assessment

The Hamilton Depression Scale-24 (HAMD-24) [28] was used to measure the severity of depressive symptoms. The Childhood Trauma Questionnaire Short Form (CTQ-SF) was used to assess the childhood trauma [29], including five subscales: Emotional Abuse (EA), Physical Abuse (PA), Sexual Abuse (SA), Physical Neglect (PN), and Emotional Neglect (EN). Impulsivity and aggressiveness were assessed by the Barratt Impulsiveness Scale (BIS-10) [30] and the Buss–Perry Aggression Questionnaire (BPAQ) [31], respectively. The BIS-10 includes three subscales: Cognitive Impulsiveness, Motor Impulsiveness, and Non-Planning Impulsiveness. The BPAQ consists of four subscales: Physical Aggressiveness, Verbal Aggressiveness, Anger, and Hostility.

### Machine learning

#### Data pre-processing

A dataset was built using simple baseline demographic characteristics (gender, age, marital status, education level), clinical questionnaires (HAMD-24, CTQ), and cognitive function (SST, IGT). These features were used to train models and predict suicide attempts for each MDD patient. Averages filled two missing values in RT of "suicide" word. The data were normalized so that different specifications could be converted into an exact specification. Subsequently, the dataset was randomly divided into training and test sets in a ratio of 7:3.

#### Model construction and interpretability analysis

ML algorithm with Extreme Gradient Boosting (XGBoost), established with the XGBoost library in Python 3.7, was used to construct the predictive models for DSA that accurately perform classification tasks. To observe the correlation between cognitive function and suicide attempts, two feature sets were made that did not include other clinical information or biological variables. XGBoost-1 was trained with demographic characteristics (gender, age, marital status, and education level) and clinical questionnaires (HAMD-24, CTQ), but did not apply data regarding cognitive function. In contrast, XGBoost-2 was trained with the above features and included cognitive function (SST, IGT). (Table 1) During the training of models, we selected hyper-parameters by grid search using Scikit-Learn library in Python. Optimal models were constructed via stratified tenfold cross-validation, which was able to prevent over-fitting of models. The workflow of ML in this study is shown in Supplementary Fig. 1. The predictive discrimination abilities of the models were assessed using sensitivity, specificity, accuracy, PPV (positive predictive value), NPV (negative predictive value) and the area under the curve (AUC), calculated using R-Studio 4.0.3. The models' performance was compared using AUC, decision curve analysis (DCA), and net reclassification improvement (NRI). NRI is a statistical method quantifying how accurately a new model reclassifies the study population compared to other models. The AUCs of the two models were compared with the Delong test using MedCalc 19.0.7.

Additionally, we added the locally explanatory technique SHAP (Shapley Additive Explanations) to explain the optimal model. SHAP is a game-theoretic approach explaining the output of ML models. It connects optimal credit allocation with local explanations using the classical SHAP values. SHAP was used to detect which features support models’ output significantly and to explain the decision-making process of models.

### Statistical analyses

Descriptive statistics, such as proportion, the sample mean, and standard deviation, were calculated by group. ANOVA F-test and Pearson's chi-squared test were used to compare continuous and categorical data, respectively, among three groups: HC, DNS, and DSA. Univariate tests were used to compare groups on net total and different scores for each block. Using the three groups, a repeated-measures analysis of variance assessed changes over the five blocks of 20 trials. DSA and DNS were compared with HC on net total score and scores of each block using Student’s t-test. The Attempter group was further sub-divided into two groups based on the lethality of suicide attempts, and IGT scores across blocks were compared across two groups using Student’s t-test. The significance level was set at *p* < 0.05. All statistical analyses were conducted in SPSS 25.0.

## Results

Descriptive statistics and comparison analyses among DSA, DNS, and HC are shown in Table 1. These analyses were considered descriptive and were not adjusted for other covariates.

### Association between depression/suicide attempts and performance of IGT

**Fig. 1:**
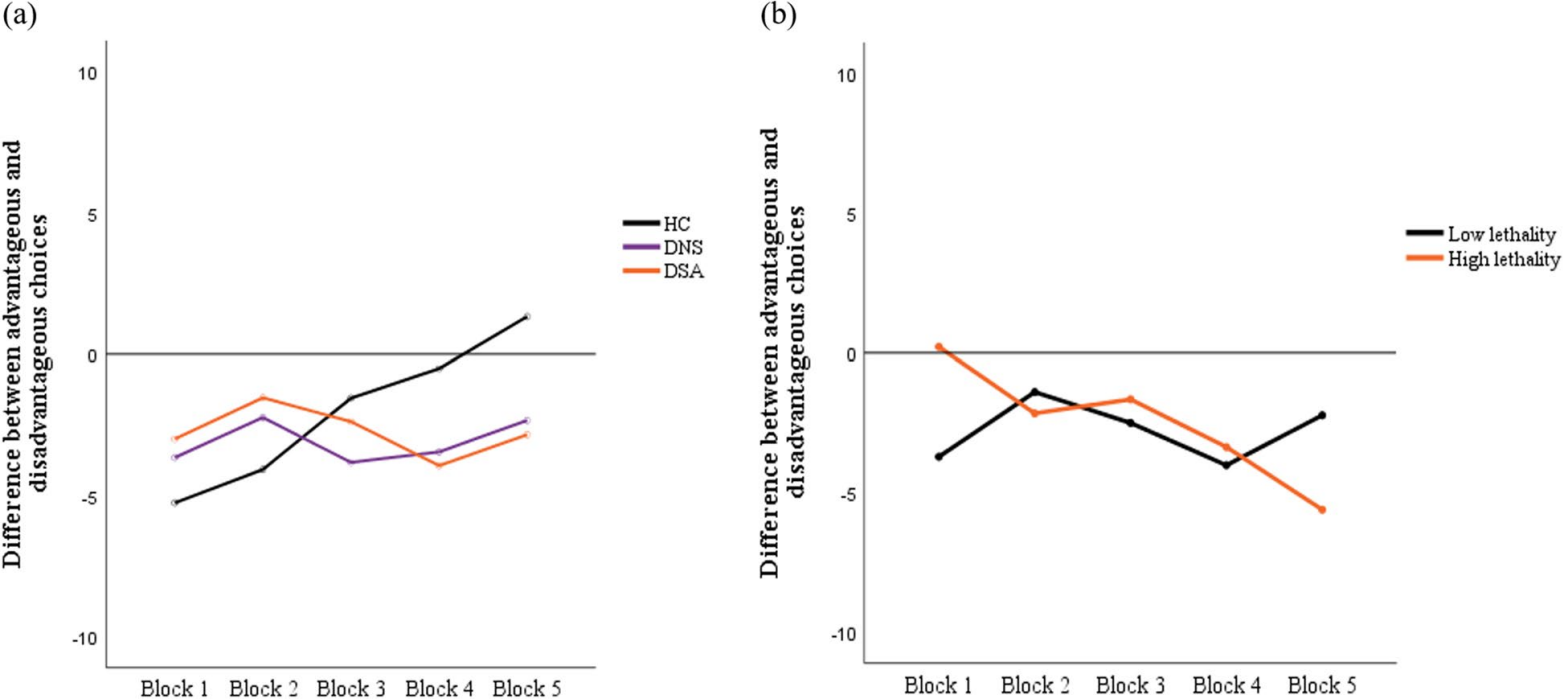
Performance of IGT. **a** Comparison of IGT scores by blocks among three groups: healthy controls (HC), depressed non-attempts (DNA) and depressed suicide attempters (DSA). **b** Comparison of IGT scores by blocks among two groups divided by the lethality of suicide attempts: low and high lethality. *DSA: depressed suicide attempter, DNS: depressed non-suicide attempter, HC: healthy control, IGT: IOWA gambling task

Table 2 and Fig. 1 show the results of the IGT score analyses. The total net scores and block scores of the three groups were all negative.

**Table 2:** Performance of IGT across all groups

	**Group**			**Group comparisons**							
	HC* (*N* = 98)	DNS* (*N* = 61)	DSA* (*N* = 52)	HC* vs DNS* vs DSA*						DNS* + DSA* vs HC*	
	**Mean(SD)**	**Mean(SD)**	**Mean(SD)**	**F**	**d.f**	***p*-value**	HC* vs DNS*	HC* vs DSA*	DNS* vs DSA*	**t**	***p*-value**
							***p*-value**	***p*-value**	***p*-value**		
IGT* performance											
Block1(Trials 1–20)	-5.27 (6.725)	-3.8 (7.269)	-3 (6.669)	2.056	2	0.131	0.195	0.056	0.54	1.935	0.054
Block2(Trials 21–40)	-4.08 (7.896)	-2.47 (8.848)	-1.54 (7.868)	1.818	2	0.165	0.229	0.071	0.549	1.813	0.071
Block3(Trials 41–60)	-1.55 (9.079)	-4.07 (9.072)	-2.38 (7.118)	1.583	2	0.208	0.077	0.574	0.305	-1.452	0.148
Block4(Trials 61–80)	-0.51 (9.465)	-3.67 (8.471)	-3.94 (7.092)	3.782	2	0.024	0.027	0.022	0.867	-2.751	0.006
Block5(Trials 81–100)	1.33 (9.877)	-2.34 (9.345)	-2.85 (9.712)	4.261	2	0.015	0.023	0.013	0.783	-2.913	0.004
Net total score	-10.06 (34.148)	-16.53 (33.422)	-13.73 (28.883)	0.755	2	0.471	0.229	0.514	0.652	-1.145	0.254
PartA(Trails 1–40)	-9.35 (12.315)	-6.27 (13.956)	-4.54 (12.221)	2.666	2	0.072	0.143	0.029	0.476	2.199	0.029
PartB(Trails 41–100)	-0.735 (25.015)	-10.03 (20.881)	-9.17 (19.571)	3.838	2	0.023	0.018	0.031	0.916	-2.775	0.006

^*^DSA Depressed suicide attempter, *DNS* Depressed non-suicide attempter, *HC* Healthy control, *IGT* Iowa Gambling Task

As indicated by Table 2, the scores of Block 4 (*F* = 3.782, *p* = 0.024), Block 5 (*F* = 4.261, *p* = 0.015) and Part B (*F* = 3.838, *p* = 0.023) differed significantly among the three groups, while IGT net total score did not (*F* = 0.755, *p* = 0.471). Post hoc testing showed that HC showed a higher preference for disadvantageous decks in Part A (*p* = 0.029) compared to DSA and advantageous decks in Part B compared to DSA (*p* = 0.031), and compared to DNS (*p* = 0.018). The scores of Block 4 (*t* = -2.751, *p* = 0.006), Block 5 (*t* = -2.913, *p* = 0.004), Part A (*t* = 2.199, *p* = 0.029) and Part B (*t* = -2.775, *p* = 0.006) differed significantly between DSA together with DNS and HC, as indicated by the results of the Student’s t-test.

As Fig. 1-a shows, three groups began by choosing from the disadvantageous decks. HC groups showed a preference for the disadvantageous decks initially, but they gradually turned to advantageous decks. However, DNS and DSA groups showed no evidence of improvement as the task progressed. To quantify the learning effects of three groups during the task, repeated measures ANOVA was used to compare IGT performance across blocks. Repeated measures ANOVA among three groups revealed a significant learning effect from the first to the fifth block of the IGT (*F* = 4.198, d*F* = 4; *p* = 0.003), and further revealed a significant effect of group-by-blocks interaction (*F* = 8.000, dF = 8; *p < *0.001). When age and gender were added as covariates in the model, the effect of group-by-blocks interaction remained significant (*F* = 4.451, dF = 8; *p < *0.001), while no significant difference was found in the learning effect (*F* = 1.603, dF = 4; *p* = 0.175). The interaction effect indicated that the scores of Block 4 and Block 5 were significantly higher than those of Block 1 (*p < *0.001) and Block 2 (*p < *0.001) in HC. However, no significant difference was found between the DNS and DSA groups. Repeated measures ANOVA between DSA together with DNS revealed a significant learning effect group-by-blocks interaction (*F* = 9.131, dF = 4; *p < *0.001) but not in difference scores across blocks (*F* = 2.158, dF = 4; *p* = 0.075).

Attempters were divided into two groups by lethality of suicide attempts (high lethality versus low lethality). As shown in Fig. 1-b, we found a tendency for the high lethality group to show a poor learning effect across the blocks compared to the low lethality group, but this difference did not reach statistical significance.

Performance and Interpretability of Machine Learning Classifiers (Sec2).

**Fig. 2:**
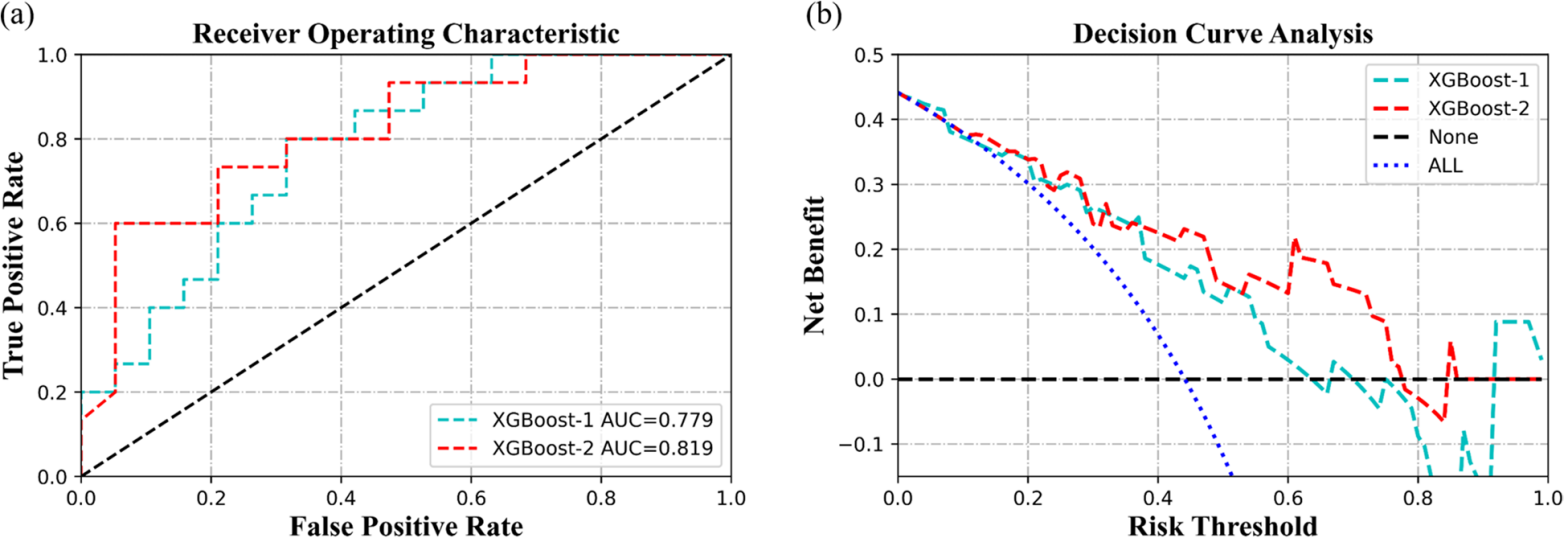
The receiver operating curve and decision curve analysis of suicide attempts predictive model, the performance of Xgboost-1 and XGBoost-2 used on testing set. *AUC: area under the curve

The dataset was randomly divided into two groups: training set (70% of 79 subjects, 37 DSA) and test set (30% of 34 subjects, 15 DSA). As shown in Table 3 and Fig. 2, XGBoost-1, trained using demographic characteristics and clinical questionnaires, showed AUC with 0.779 (95% confidence intervals (CI): 0.627,0.934) in test set. After adding cognitive function (SST, IGT), XGBoost-2 achieved AUC with 0.819 (95% CI: 0.675,0.964). The Delong test showed the AUCs of XGBoost algorithm had improved by 0.040 (*p* = 0.44). Additionally, the specificity, accuracy, PPV, and NPV had ascended 5.3%, 2.9%, 4.9%, and 1.4%, respectively. (Table 3) DCA showed that the diagnostic efficiency and net benefit of XGBoost-2 is better than that of XGBoost-1 when the risk threshold is set in the range of 0.38 to 0.86. XGBoost-2 resulted in continuous reclassification improvement compared to XGBoost-1 with NRI of 5.3%.

**Table 3:** The testing result of XGBoost classification algorithm on suicide attempts among patients with MDD

	**Sensitivity**	**Specificity**	**Accuracy**	AUC*	PPV*	NPV*
XGBoost-1	0.600 (0.323,0.837)	0.737 (0.488,0.909)	0.677 (0.495,0.826)	0.779 (0.627,0.934)	0.643 (0.351,0.872)	0.700 (0.457,0.881)
XGBoost-2	0.600 (0.323,0.837)	0.790 (0.544,0.940)	0.706 (0.525,0.849)	0.819 (0.675,0.964)	0.692 (0.386,0.909)	0.714 (0.478,0.887)

^*^AUC Area under the curve, *PPV* Positive predictive value, *NPV* Negative predictive value

**Fig. 3:**
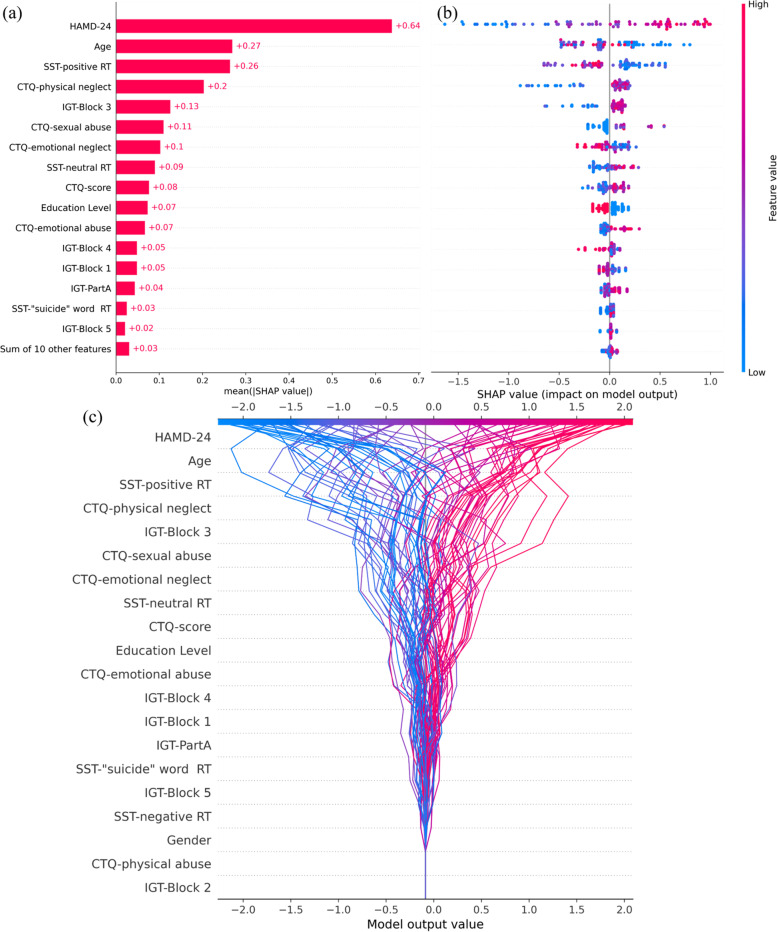
The pre-selected feature sets of the optimal model were evaluated through SHAP. **a** Features are listed in descending order according to contributions for the XGBoost-2 in predicting suicide attempts. **b** The feature effects on identifying suicide attempts. The color indicates the values of the features from high to low. The horizontal location shows whether the effect of the value leads to the prediction of suicide attempts. Each point is a SHAP value for a case and a feature. **c** The decision plot of XGBoost-2 predicting suicide attempts. Each line represents a case. From the bottom of the plot to the top, SHAP values for each feature are added to the base value of model, and each line strikes the x-axis at its corresponding observation’s predicted value to obtain prediction results. *IGT: IOWA gambling task, SST: suicide stroop task, HAMD-24:Hamilton Depression Scale-24 items, CTQ: Childhood Trauma Questionnaire

SHAP was used to evaluate and analyze further the optimal feature set for XGBoost models, shown in Fig. 3 and Supplementary Fig. 2. HADM-24 was the top predictive factor affecting the classification of XGBoost-2, followed by age and positive RT. Analyzing the results of cognitive tasks, positive RT and neutral RT in SST, and the performance of Block 3 in IGT were the top three predictive factors. High scores on HAMD-24, younger age, and shorter RT for positive words are strongly associated with suicide attempts, and this finding supports XGBoost-2 classifying them as such. Integrating the other results of cognitive function (SST, IGT) and various questionnaires (HAMD-24, CTQ) can support the XGBoost model to predict suicide attempts better.

## Discussion

This study explored the relationship between cognitive deficits and suicide attempts and established a predictive model for suicide attempts among MDD patients by ML. It yielded two findings. First, the DSA group performed more conservatively in decision-making under ambiguous conditions compared to the HC group. The DSA and DNS groups made more disadvantageous choices in risky conditions, while showing no significant improvement due to the learning effect as the task progressed, compared to HC. Second, a clinically useful predictive model for DSA was constructed, and demonstrated that cognition could help ML algorithms to predict the risk of suicide attempts in MDD patients.

In this study, we used the Iowa Gambling Task to test the performance of suicide attempters with MDD on decision-making under conditions of ambiguity and risk. We found that the DSA-only group and DSA, together with DNS, showed a more conservative performance than the HC group on decision-making in ambiguous conditions. Damasio proposed that making advantageous decisions can be learned through emotions generated by rewarding and punishing feedback based on previous decisions [32]. Previous studies demonstrated hyposensitivity to rewards and hypersensitivity to punishments in MDD patients (including patients with a history of suicide attempts) [33]. Thus, the intense negative emotional experience from punishing feedback may warn MDD away from choosing the disadvantageous decks repeatedly. Compared to HC, the poor learning effect of DSA during decision-making indicates that they are unlikely to develop the ability to balance risk versus reward elements effectively based on trial-and-error experience [34]. Statistically significant deficits in decision-making were found in both DSA only and DSA combined with DNS, but not in DNS only compared to HC, implying that suicide attempts may play an important role in decision-making. The association between MDD patients and decision-making was found in a previous study [35], but Pustilnik found a direct relationship between impaired decision-making and suicide risk. Impaired decision-making may increase suicide risk more strongly than the influence of depression [9].

Consistent with previous studies, we did not find a significant difference between DNS and DSA [10]. To further analyze DSA, the HLSA group showed a tendency toward poor learning ability compared with the LLSA group during the task; therefore, we concluded that deficits in decision-making might be associated with suicide attempt lethality, which is consistent with previous studies [34]. The small sample size of the HLSA group may be the reason why we did not find statistically significant differences between the two groups. Additionally, according to the definition, a history of suicide attempts may span over a long period of time. Therefore, during this period, decision-making may be affected by various factors. Thus, the deficits in decision-making may be detectable only among recent suicide attempters [10]. Indeed, going through a suicidal crisis recently may affect attitude towards decision-making differently than an attempt that took place in the more distant past. Accordingly, recent attempters may still have a potential wish to die [10]. In our study, however, relatively few participants (*N* = 9, 17.3%) had a history of attempted suicide within the previous week. Individuals who had suicide attempts during the previous week or exhibited high lethality behavior may visit the emergency department or require hospitalization or treatment in special units. All participants in our study, however, were recruited from the psychiatric department. These factors could have led to a failure to detect significant differences between the two subgroups among suicide attempters.

The first part of our study mainly confirmed the role of cognitive deficits of decision-making in patients with a history of suicide attempts and revealed the possibility that existence of these deficits is a critical step in the early identification of potential targets for risk evaluation and treatment. The second part of our study described the construction of a clinically useful predictive model for suicide attempts among MDD patients.

For the study, the key objectives were to construct ML models that integrate the advantages of multiple scales and observe whether cognitive function positively affects the prediction of suicide attempts. XGBoost-2 resulted from adding features of cognitive function (SST, IGT) to XGBoost-1. Our ML classifiers presented relatively good performance in the task of classifying suicide attempters in MDD patients in accordance with previous studies that used shallow or deep learning algorithms [17, 36, 37]. According to the statistical indicators, ROC curve, DCA, and NRI, when cognitive function was incorporated into XGBoost model, it exhibited improved model fit and superior predictive accuracy and improved patients net benefits, while maintaining the same level of sensitivity for DSA. The ML model can provide prediction probability for clinicians’ reference in clinical practice, but this is insufficient. Therefore, we added a local interpretation method, SHAP, to show which and how variables support models’ output significantly.

According to the SHAP value shown in Fig. 3, the most important feature for differentiating suicide attempters from MDD patients in XGBoost-2 was HAMD-24, as in previous studies [17, 38]. Additionally, cognitive function played an essential role in XGBoost-2, referring to multiple interactions. Among them, RT for positive words has the greatest contribution to the model prediction of DSA, and IGT-Block 3 and RT for neutral word are also of high importance. Furthermore, in view of the overall improvement of model performance, other features in SST and IGT cannot be ignored owing to the sum of their feature importance.

**Fig. 4:**
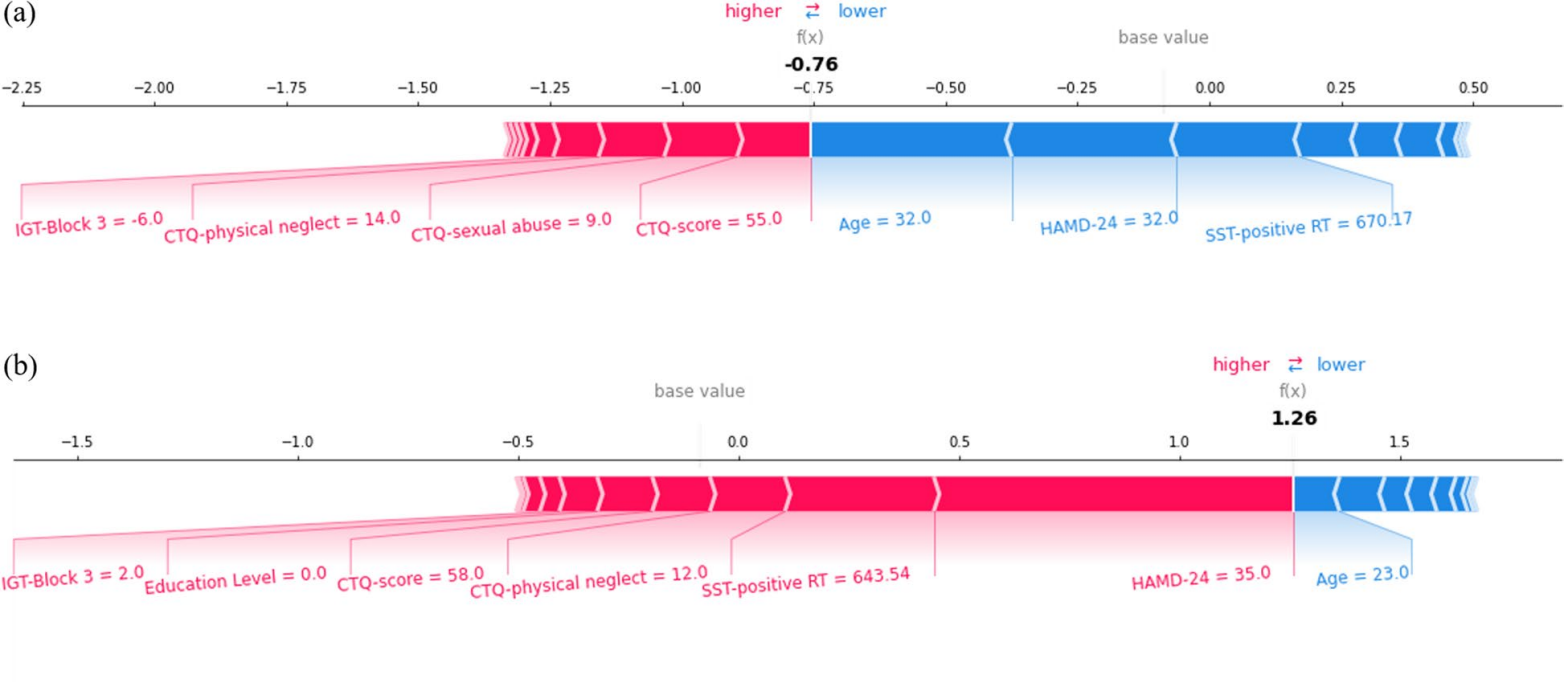
The force plot for decision process of XGBoost-2 evaluating whether two MDD patients in test set had suicide attempts. Each feature provides a SHAP value for the base value of model. The final prediction value, f (x), are obtained according to the weight of features and the processing of the machine learning algorithm. When f (x) > 0, the model considers the case as DSA, otherwise it is considered as DNS. *IGT: IOWA gambling task, SST: suicide stroop task, HAMD-24:Hamilton Depression Scale-24 items, CTQ: Childhood Trauma Questionnaire

Figure 4 displays the decision process of XGBoost-2 in two specific cases in test set. For Fig. 4a, a DNS case, due to relatively older age, low HAMD-24 score, and long RT for positive words, the model considered that this patient did not have suicide attempt. However, for this case, childhood trauma, especially sexual abuse and physical neglect could be potential factors associated with suicide attempts that deserve clinicians’ attention. For Fig. 4b, a DSA case, we can find that although young age is a risk factor for suicide, other features, including high HAMD-24 score, short RT for positive words, high CTQ score, etc., support the model to classify this case as DSA with solid confidence. Figure 3c summarizes the visual decision process of each patient in test set by XGBoost-2, which might help clinicians analyze the suicide risk of MDD patients in a more organized, accurate manner.

This study established a clinically useful predictive model for suicidality using cognitive function and clinical and demographic data through ML algorithms. One remaining crucial question is how this model might be applied in clinical practice to optimize individual patient treatment. We hypothesize that highly accurate predictive models will support important clinical decisions, such as selecting treatment options, preventive strategies, and prognosis orientations. For example, it is possible that high-risk individual patients, as predicted by the model, would benefit from specific therapies [39] or anti-suicidal drugs, such as lithium [40, 41] and clozapine [42]. Additionally, knowledge of the mechanisms of cognitive impairments may help in distinguishing patients at risk for suicide, and guide preventive strategies, such as clinically feasible cognitive evaluations and preventive interventions [43, 44]. Based on the visualization methods of the ML model used in this study, clinicians can clearly identify the suicide risk factors for each MDD patient and intervene in a targeted manner.

Several studies recently utilized ML techniques to predict treatment response using functional brain scans and cognitive data [45, 46]. Therefore, we hope that our study could provide fruitful future directions for suicide prediction research. During our next step within this research endeavor, we will consider including more comprehensive patient clinical information, such as genetic, neuroimaging, and biological variables, and expanding the data scale to focus on constructing a robust predictive model for suicide attempts. A wider variety of shallow and deep learning algorithms should be applied to this promising research. Future studies should also develop novel suicide stratification algorithms to translate results from predictive models into clinical practice, and individualize treatments for patients at high risk of attempting suicide.

## Limitations

There are some limitations of our study that merit discussion. First, the study sample is small and drawn only from patients with MDD due to the strict inclusion and exclusion criteria, which may not fully represent the population of suicide attempters and may explain why the performance of the IGT did not align with the results of previous studies. Thus, we chose XGBoost to complete the task of models’ construction, because when processing data with small samples and multiple features, the ensemble ML models are often better than other shallow ML models or even neural networks. Second, cognitive function involves multiple components [47], and our results may not reflect the influence of other cognitive deficits, such as attention, executive function, memory, and working memory, which were not included in the predictive model. Third, the development of suicide risk is complex, involving contributions from biological (including genetics), psychological (such as certain personality traits), clinical (such as comorbid psychiatric illness), social and environmental factors. Future studies are needed to construct more comprehensive predictive models with different types of factors and expand data scale. The current study, therefore, serves as a proof-of-concept. Future longitudinal studies with larger samples and more comprehensive risk factors are needed to replicate our findings and determine the causal links between cognition and suicide.

## Conclusions

In summary, our results demonstrate that cognitive deficits may serve as an important risk factor to predict suicide attempts in patients with MDD. Combined with other demographic characteristics and attributes drawn from clinical questionnaires, cognitive function can improve the predictive effectiveness of the ML model. Additionally, explanatory ML models can help clinicians detect specific risk factors for each suicide attempter within MDD patients. These findings may be helpful for clinicians to detect those at high risk of suicide attempts quickly and accurately, and help them make proactive treatment decisions.

## Supplementary Information

**Additional file 1:** **Supplementary Figure 1.** The workflow of ML in this study is as follows. A dataset was built using baseline demographic characteristics (gender, age, marital status, education level), clinical questionnaires (HAMD-24, CTQ), and cognitive function (SST, IGT). For the missing data problem, averages filled two missing values in RT of "suicide" word, and no other features were missing. Then, the data were normalized to the range of 0-1 by calling the MinMaxScaler function from sklearn.preprocessing module in Python 3.7, so that different specification could be converted into an exact specification. Subsequently, the dataset was randomly divided into training and test sets in a ratio of 7:3 by calling the train_test_split function from sklearn.model_selection module. Because this is a small sample size study, and we wanted to observe the importance of all features in assessing the suicide risk of MDD patients, we did not perform feature selection to reduce the dimension. In the training phase of the ML models, the XGBoost-1 and XGBoost-2 were constructed with different feature sets but the same workflow. We selected hyper-parameters by grid search and 10-fold cross-validation using the Scikit-Learn library, and in this work, we set the n_estimators as a range of 1-200 and the max_depth as a range of 1-10. Then we used the independent test set to validate the performance of the models and got the final results. **Supplementary Figure 2.** The pre-selected feature sets of XGBoost 1 were evaluated through the Shapley values. Model 1 predicts whether the patients with MMD as suicide attempter mainly according to HAMD-24. Other characteristics affected the judgment of the model to varying degrees.

## Data Availability

The datasets generated and/or analysed during the current study are not publicly available but are available from the corresponding author on reasonable request.
