# Supplementary material for: Can cognition help predict suicide risk in patients with major depressive disorder? A machine learning study
Source: BMC Psychiatry. 2022 Sep 1;22:580. doi: 10.1186/s12888-022-04223-4 (PMC9434973; doi:10.1186/s12888-022-04223-4)

**Supplementary Figure 1**: The workflow of ML in this study is as follows. A dataset was built using baseline demographic characteristics (gender, age, marital status, education level), clinical questionnaires (HAMD-24, CTQ), and cognitive function (SST, IGT). For the missing data problem, averages filled two missing values in RT of "suicide" word, and no other features were missing. Then, the data were normalized to the range of 0-1 by calling the MinMaxScaler function from sklearn.preprocessing module in Python 3.7, so that different specification could be converted into an exact specification. Subsequently, the dataset was randomly divided into training and test sets in a ratio of 7:3 by calling the train_test_split function from sklearn.model_selection module. Because this is a small sample size study, and we wanted to observe the importance of all features in assessing the suicide risk of MDD patients, we did not perform feature selection to reduce the dimension. In the training phase of the ML models, the XGBoost-1 and XGBoost-2 were constructed with different feature sets but the same workflow. We selected hyper-parameters by grid search and 10-fold cross-validation using the Scikit-Learn library, and in this work, we set the n_estimators as a range of 1-200 and the max_depth as a range of 1-10. Then we used the independent test set to validate the performance of the models and got the final results.


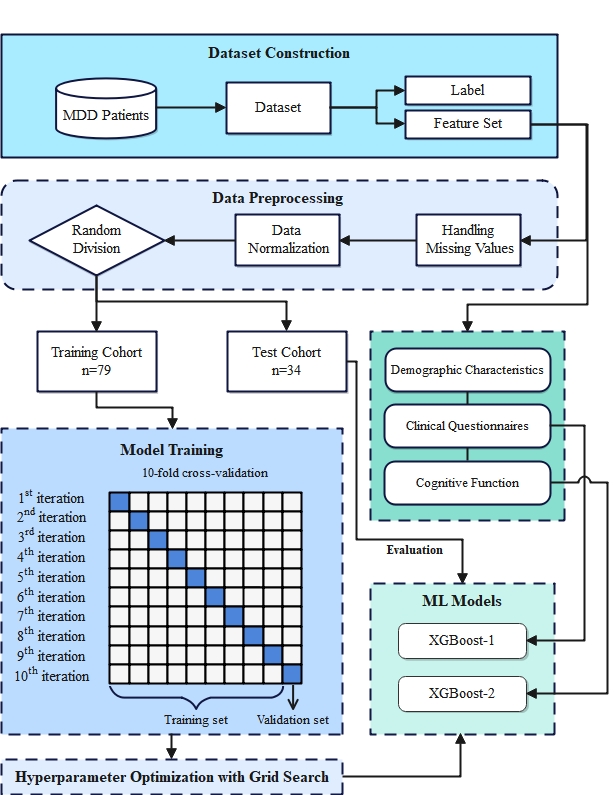


**Supplementary Figure 2**: The pre-selected feature sets of XGBoost 1 were evaluated through the Shapley values. Model 1 predicts whether the patients with MMD as suicide attempter mainly according to HAMD-24. Other characteristics affected the judgment of the model to varying degrees.


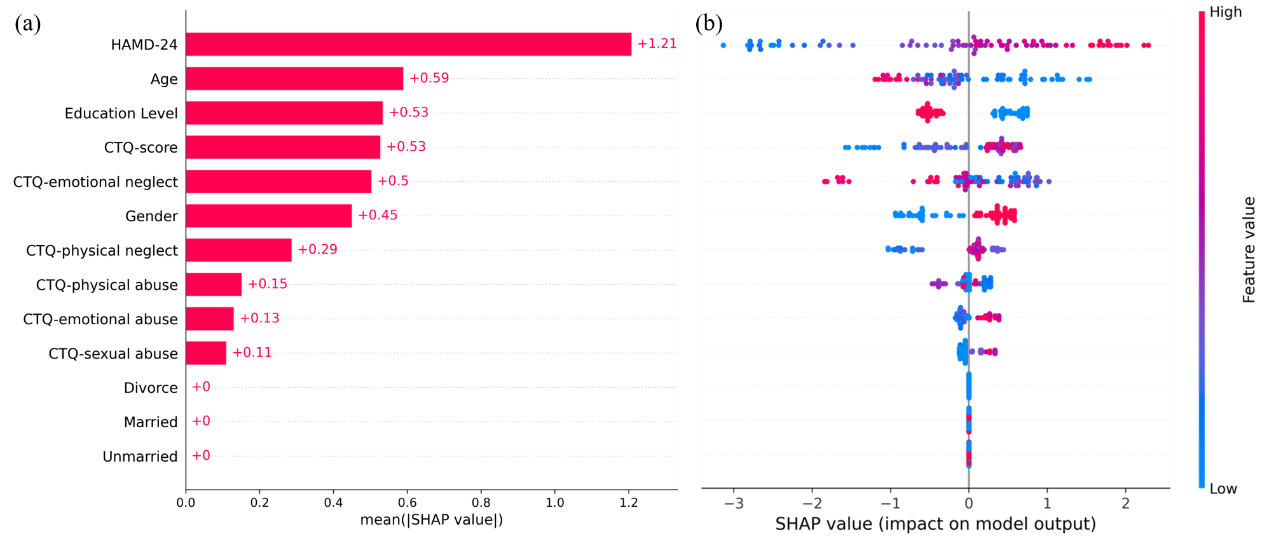

Supplement: Supplementary file 1 — Additional file 1: Supplementary Figure 1. The workflow of ML in this study is as follows. A dataset was built using baseline demographic characteristics (gender, age, marital status, education level), clinical questionnaires (HAMD-24, CTQ), and cognitive function (SST, IGT). For the missing data problem, averages filled two missing values in RT of "suicide" word, and no other features were missing. Then, the data were normalized to the range of 0-1 by calling the MinMaxScaler function from sklearn.preprocessing module in Python 3.7, so that different specification could be converted into an exact specification. Subsequently, the dataset was randomly divided into training and test sets in a ratio of 7:3 by calling the train_test_split function from sklearn.model_selection module. Because this is a small sample size study, and we wanted to observe the importance of all features in assessing the suicide risk of MDD patients, we did not perform feature selection to reduce the dimension. In the training phase of the ML models, the XGBoost-1 and XGBoost-2 were constructed with different feature sets but the same workflow. We selected hyper-parameters by grid search and 10-fold cross-validation using the Scikit-Learn library, and in this work, we set the n_estimators as a range of 1-200 and the max_depth as a range of 1-10. Then we used the independent test set to validate the performance of the models and got the final results. Supplementary Figure 2. The pre-selected feature sets of XGBoost 1 were evaluated through the Shapley values. Model 1 predicts whether the patients with MMD as suicide attempter mainly according to HAMD-24. Other characteristics affected the judgment of the model to varying degrees. [file 12888_2022_4223_MOESM1_ESM.docx]
